# An Integrated Framework to Model Cellular Phenotype as a Component of Biochemical Networks

**DOI:** 10.1155/2011/608295

**Published:** 2011-11-29

**Authors:** Michael Gormley, Viswanadha U. Akella, Judy N. Quong, Andrew A. Quong

**Affiliations:** ^1^Department of Cancer Biology, Kimmel Cancer Center, Thomas Jefferson University, Bluemle Life Sciences Building, 233 S. 10th Street, Philadelphia, PA 19107, USA; ^2^School of Biomedical Engineering, Science and Health Systems, Drexel University, 3141 Chestnut Street, Philadelphia, PA 19104, USA; ^3^American Association for Cancer Research, Cancer Discovery, 615 Chestnut Street, Philadelphia, PA 19106, USA

## Abstract

Identification of regulatory molecules in signaling pathways is critical for understanding cellular behavior. Given the complexity of the transcriptional gene network, the relationship between molecular expression and phenotype is difficult to determine using reductionist experimental methods. Computational models provide the means to characterize regulatory mechanisms and predict phenotype in the context of gene networks. Integrating gene expression data with phenotypic data in transcriptional network models enables systematic identification of critical molecules in a biological network. We developed an approach based on fuzzy logic to model cell budding in *Saccharomyces cerevisiae* using time series expression microarray data of the cell cycle. Cell budding is a phenotype of viable cells undergoing division. Predicted interactions between gene expression and phenotype reflected known biological relationships. Dynamic simulation analysis reproduced the behavior of the yeast cell cycle and accurately identified genes and interactions which are essential for cell viability.

## 1. Background 

Efforts to develop therapeutics for complex disorders such as cancer, infectious disease, and autoimmune disease require an understanding of the specific pathways through which networks of molecular interactions influence cellular function. Due to the complexity of biochemical pathways, a combinatorially large number of experiments that can simultaneously measure the changes in gene or protein expression such as a microarray or an LCMS-based proteomics are required in order to fully characterize normal and disease-producing mechanisms [1]. An iterative approach, using computational biology to complement high-throughput experimentation, may increase the efficiency by which data can be gathered by eliminating redundant or irrelevant experiments and suggesting hypotheses to build optimally upon current knowledge [2–4]. Development of gene expression microarray platforms enables the collection of expression data on a genome-wide scale sufficient for the derivation of gene-gene interactions and reverse engineering of system's scale models of gene networks [5, 6]. However, computational models of biological systems often disregard cellular phenotype data. Phenotype should be explicitly incorporated in computational gene network models to contextualize perturbations according to their effect on the desired change in cellular phenotype. This not only allows for a seamless coupling between computation and experimentation but also enables a guided search to identify molecules, complexes, and pathways that regulate disease-specific processes such as migration, proliferation, differentiation, or cell death [2, 4]. 

A range of methodologies have been developed to reverse engineer transcriptional networks from expression data. The choice of an appropriate modeling method is dependent on the scale of the modeled system, quality of data, and availability of prior knowledge. Dimension reduction approaches such as principal component analysis or partial least squares regression can be applied to identify correlated patterns of expression that can be viewed as abstract representations of pathways or coregulated molecules [6]. These methods are well suited for poorly characterized systems as they are designed to operate on high-dimensional datasets and require no prior knowledge. However, it can be difficult to predict changes in cellular phenotype based on relationships observed in transformed space with reduced dimensionality. In contrast, differential equation-based models can be used to approximate highly specific spatial and temporal characteristics of gene networks [5]. Applicability of differential equation-based methods is limited by the extensive amount of prior knowledge required, sensitivity to noisy data, and computational cost. With these constraints, modeling by the use of differential equations is confined to smaller, well-defined systems for which precise quantitative data is available. Logic-based models, such as Boolean networks and fuzzy logic, are generated by the identification of simple relationships between variables in a discretized measurement space. In this manner, logic-based models compromise specificity for computational tractability and robustness to noisy data. Identification of relevant input data and the relationship between input and output variables can be defined based on prior knowledge [7] or inferred in a data-driven manner [8, 9]. As such, logic-based methods can be applied to analyze biological systems that are poorly defined. Additionally, these methods provide a framework to incorporate quantitative and qualitative information such as linguistic and graphical representations of biological systems [10]. Although the simplicity of Boolean network models is attractive, binary representation lacks the dynamic range to sufficiently model biological complexity [11]. Of the methods described above, fuzzy logic-based approaches offer the proper balance between computational cost and biological interpretability for the specification of mechanistic transcriptional models on a genome-wide scale. 

Fuzzy logic-based biological network models can be viewed as a directed graph, in which nodes represent genes, proteins, phenotypes, or other measurable variables and edges symbolize direct or indirect interactions between variables. Fuzzy models are derived by a process including fuzzification of variables, model estimation, and evaluation of the model against observed data. Fuzzification is the process by which continuous variables are converted into sets of discrete variables that describe the degree to which the values fit one or more classes [12]. For example, gene expression values can be represented in three fuzzy sets: low, medium, and high expression. Membership functions describe the relationship from continuous to fuzzy space by transforming each value to a number in the interval [0,1]. Values closer to one indicate a higher degree of membership. Membership functions can be defined arbitrarily or with the use of optimization methods [13]. Additionally, membership functions can overlap, such that a continuous variable maps to multiple fuzzy sets. These properties reflect the subjective nature of fuzzy logic, provide robustness to noisy data, and enable intermediate activation states to be modeled [13]. Model estimation includes the identification of network topology that describes which variables interact and specification of the nature of the relationships (i.e., activation, inhibition, etc.). This information is used to define a set of rules that map between input and output data in fuzzy space. Rules can be described linguistically as if-then relationships between variables. For example, a positive regulatory relationship between genes A and B can be described by the following rules: if expression of gene A is high, then expression of gene B is high; if expression of gene A is medium, then expression of gene B is medium; if expression of gene A is low, then expression of gene B is low. Modeling of transcriptional networks is complicated by the observation that gene expression and activation is often controlled by multiple regulatory genes. In fuzzy logic, the influence of multiple inputs can be summed to derive the state of the output variable in fuzzy space. Prior to model evaluation, predicted output values must be defuzzified from fuzzy space back to continuous space. Defuzzification functions are defined dependent on the membership functions used for fuzzification. Models can be evaluated qualitatively by comparing predicted interactions with regulatory mechanisms described in the literature and quantitatively by calculating the fit of defuzzified expression values of output nodes with observed data. Best fit models are identified by exhaustive search [9] or by the use of optimization schemes, such as genetic algorithms [8], to explore the solution space. 

Evaluation of reverse engineering algorithms is facilitated by the study of model organisms in which extensive prior knowledge regarding gene regulatory and cell signaling mechanisms has been collected. Cell cycle regulation in *Saccharomyces cerevisiae *has been thoroughly described through the use of genetic manipulation [14], gene expression microarray [15] and computational modeling [5, 16, 17]. The goal of the present work is to extend on these ideas to infer molecular pathways or networks and then use this inference to predict cellular phenotype; this is not meant to provide a detailed biochemical computational model, and we do not suggest that this should replace any of the well-known models that include details neglected here such as posttranslational modification of proteins. 

 In previous work, we demonstrated the feasibility of using fuzzy logic to derive gene regulatory models from time series gene expression microarray data of twelve genes associated with cell cycle control in yeast [9]. In this study, we have extended this framework to model gene-phenotype as well as gene-gene regulatory relationships and to represent functionally homologous genes. Towards this end, we generated a gene regulatory model consisting of seventeen cell cycle-related genes and a node representing the fraction of budding cells as a morphological indicator of cell cycle progression. Evaluation of model predictions indicated that the model accurately captures regulatory events in cell cycle progression as well as known biological relationships between genes and between genes and phenotype. Investigation of the dynamic behavior of the model enabled highly accurate identification of genes essential for cell viability as well as the prediction of indirect gene-gene interactions. The methodology developed in this study provides a proof of concept for modeling regulatory influences of gene expression on phenotype in a semiquantitative manner and supports the application of fuzzy logic-based modeling in the investigation of more complex cellular mechanisms in higher organisms. 

## 2. Results

### 2.1. Yeast Cell Cycle Model

A simplified model of the cell cycle in *Saccharomyces cerevisiae* was generated using a data-driven model generation process to identify putative gene-gene and gene-phenotype interactions on the basis of gene expression microarray time series data [15]. The set of seventeen genes included in the model have been identified as central regulators of cell cycle progression [17, 18]: cyclins (*CLN1*-*3*, *CLB1*-*2*, *CLB5*-*6*), cyclin-dependent kinases (*CDC28*), transcription factors (*MCM1*, *MBP1*, *Swi4*, *Swi5*, *Swi6*), as well as cell cycle activating (*CDC6*) and inhibiting (*SIC1*, *CDH1*, *CDC20*) genes (Table 1). In order to describe the effect of gene expression on phenotype, we also included data describing the fraction of budding cells observed throughout the time series. Budding begins at the G1/S phase transition and continues through the end of M phase [19]. As such, the fraction of budding cells is a morphologically observable indicator of the number of cells in a population that are actively undergoing cell division. 

**Figure 1:**
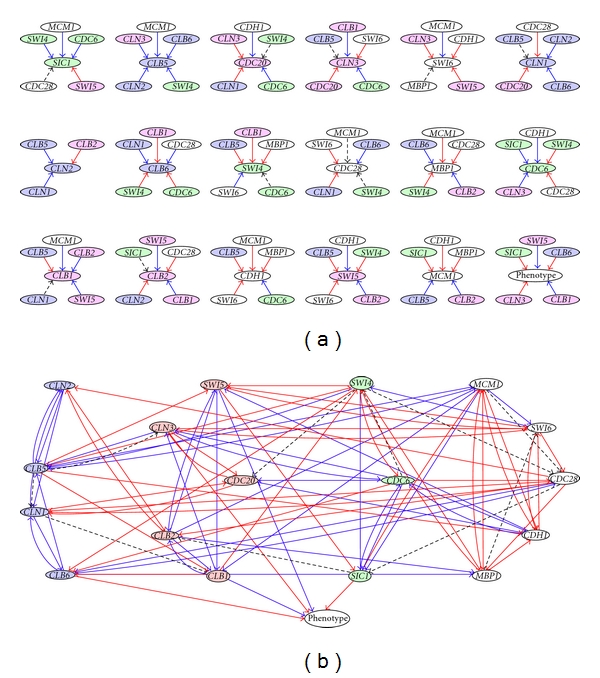
Graphical depictions of best fit models identified using the fuzzy logic model-fitting procedure. Nodes representing genes are colored according to the phase of the cell cycle in which they reach peak expression. (Blue: G1 expression, red: M expression, green: M/G1 expression, white: phenotype/expression independent of cell cycle progression.) Edges between nodes represent inferred physical/genetic/indirect interactions between genes and gene products. Blue lines indicate positive interactions. Red lines indicate negative interactions. Dashed lines indicate biphasic interactions. (a) Best fit models for expression of each gene and the fraction of budding cells (phenotype) were identified by exhaustive search through the solution space using fuzzy logic. (b) Network diagram of integrated best fit models. Nodes are organized according to the phase of the cell cycle in which they reach peak expression.

**Figure 2:**
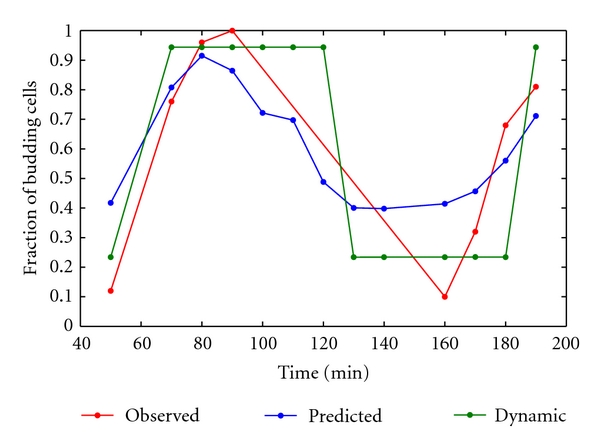
Observed and predicted fraction of budding cells at different time points in cell cycle progression. The red line and associated data points indicate the observed fraction of budding cells. The blue line indicates the fraction of budding cells predicted on the basis of gene expression. The green line indicates the fraction of budding cells predicted at convergence of the dynamic model. The fuzzy rules used to predict the fraction of budding cells are as follows: *SIC1 *(3,3, 1); *CLN3 *(3,1, 1); *CLB6 *(3,3, 1); *CLB1 *(1,1, 3); *SWI5 *(1,1, 3).

A fuzzy logic framework was implemented to derive regulatory relationships between genes and between genes and the fraction of budding cells from data collected at different time points throughout the cell cycle. Gene expression data used for model generation and evaluation were obtained by synchronizing cells by one of three different methods: *α* factor, elutriation, and *CDC15*-based synchronization [15]. Data obtained from the *CDC15*-based synchronization method were used in this study, as this is the only dataset for which phenotype data was available. Within the fuzzy logic framework, an exhaustive search was used to identify the best fitting relationships from the set of all potential interactions (i.e., gene A interacts with gene B) and all possible fuzzy rules that relate the state of input nodes to output nodes. The coefficient of determination (COD) was used to calculate the fitness of the data generated models by comparing the predicted gene expression values or fraction of budding cells with experimentally observed measurements. High values indicate that the identified models accurately predict the magnitude of expression values over the measured time points (Table 2). Figures 1(a) and 1(b) depict the best fit models for each node and the integrated network model, respectively.  Figure 2 is a plot of the observed and predicted fraction of budding cells over time derived from the best fit phenotype model. The predicted phenotype largely reproduces the observed fraction of budding cells over the time series. Exceptions to the close fit between predicted and observed phenotypes are located at time points at which the observed fraction of budding cells is minimal (e.g., 50 min, 140–170 min). Nevertheless, high correlation between predicted values and observed measurements over a majority of the time series suggest that the model built on simplified relationships in fuzzy space sufficiently captures the complex, multivariate relationships between genes and phenotype (Table 2). 

A number of the genes included in the cell cycle model are known to be functionally homologous. *CLN1* and *CLN2*, as well as *CLB1/CLB2* and *CLB5/CLB6*, have a high degree of sequence similarity (57 percent identity [21]). Additionally, yeast strains with deletions of either *CLN1* or *CLN2* remain viable and divide normally, suggesting that these proteins are interchangeable parts in the cell cycle machinery [15, 22]. In order to accurately model redundancy, we manually replaced the nodes representing these functionally homologous genes with a node corresponding to the maximum expression value of the homologous pair. The maximum operator is a common representation of a logical union and a reasonable approximation of functionally homologous genes in the fuzzy logic framework described above. Comparison of model fitness with and without the homologous gene representation supports that this is an appropriate method to account for functional homology (see Table  1 in Supplementary Material available online at doi:10.1155/2011/608295). 

### 2.2. Dynamic Modeling of Cell Cycle Progression for In Silico Prediction of Essential Genes

Gene and phenotype-specific fuzzy models were integrated into a composite model to investigate the dynamic behavior of the system as an interdependent network (Figure 1(b)). Physiologically relevant initial conditions for each node were derived from expression values at each time point and the observed fraction of budding cells at each time point in the *CDC15* dataset [15]. The state of each node was iteratively updated based on the state of its immediate input genes and the inferred fuzzy rules describing the relationships between nodes. In effect, expression data from each time point serves as a separate set of initial conditions to investigate the dynamic behavior of the model. Iteration continued until the state of the model remained relatively constant.  Figure 2 shows the predicted fraction of budding cells at each time point at convergence of the dynamic model. The model converges to two local minima corresponding with either a high or low fraction of budding cells. Notably, yeast cell cycle has been described through experimentation as an oscillation between the “alternative, self-maintaining states” of  G1 and S/M phase [20]. As cell budding is absent in G1 and maximal in S/M phase, the behavior of the model matches this hypothesis of yeast cell cycle dynamics developed from experimental investigation. In addition, the timing of the transitions between local minima is similar to the observed changes in the fraction of budding cells. 

Gene knockout is a powerful method for characterization of gene function and identification of essential genes necessary for cell viability. Using the dynamic model, we predicted the identity of essential genes by fixing the state of the corresponding node to a low expression profile to simulate gene knock-out. Viability of *in silico *gene knockout models was determined by observing the predicted fraction of budding cells. The phenotype of viable models closely approximates the oscillatory behavior of the experimentally observed fraction of budding cells over time (Figure 2). Inviable models were identified by convergence to a constant fraction of budding cells over time, indicating cell cycle arrest. The predicted viability of *in silico* gene knock-out models was compared to experimental observations from a systematic investigation of single gene deletions in yeast under normal growth conditions [14]. In this manner, in the absence of a true validation set of independent gene expression and phenotype data, independent experimental observations were used to evaluate the validity of our model.  Table 3 summarizes the correlation between predicted and observed viability. Overall, phenotypes resulting from knockout of fourteen of seventeen genes were correctly predicted. Accurate identification of essential genes with simulation of gene knockouts *in silico *indicates the validity of the inferred yeast cell cycle model and demonstrates the value of fuzzy logic models in hypothesis generation. 

**Figure 3:**
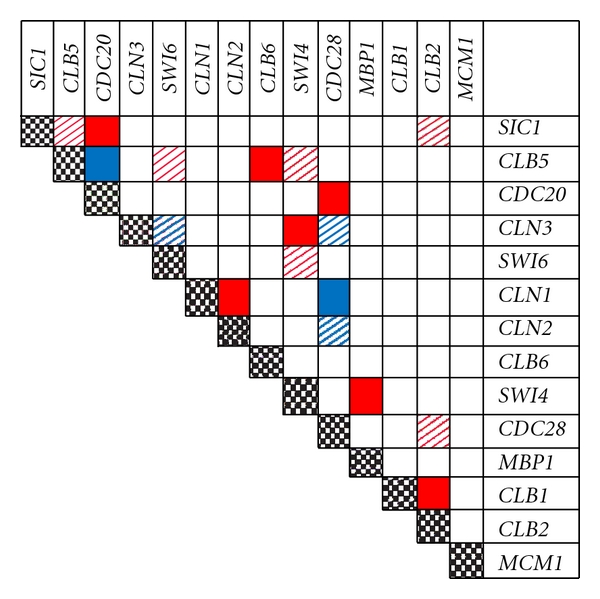
*In silico* gene knock-down models predict the viability of synthetic lethal and synthetic rescue double-gene knock-out experiments. The outcomes of double-gene knock-out experiments were obtained from publications compiled by the *Saccharomyces* Genome Database [20]. Pairs of genes that form synthetic phenotypes are identified by the color of the squares at the intersection of rows and columns. Experimentally observed synthetic lethal and synthetic rescue mutations are indicated by red and blue squares, respectively. The predicted outcome of double-gene knockouts is indicated by the pattern of the squares. Correct and incorrect predictions are marked with filled and diagonally hashed squares, respectively. Seven out of thirteen synthetic lethal and two out of five synthetic rescue phenotypes are correctly predicted.

Double-gene knockouts can be used to discover genes with physical or genetic interactions that are essential for proper cellular function. Synthetic lethals are observed when a double knockout induces lethality and both single gene knockouts are viable. Conversely, synthetic rescue is observed when a double knockout of an essential and nonessential gene alleviates the lethality of the essential gene knockout. As an additional challenge to our dynamic yeast cell cycle model, we evaluated the predicted viability of double-gene knockouts against experimentally identified synthetic phenotypes. Experimental observations were obtained from publications compiled in the *Saccharomyces* Genome Database [23]. We limited the analysis to the fourteen genes for which viability is correctly predicted with *in silico *single-gene knockouts. Among these genes, thirteen gene pairs have been observed to induce a synthetic lethal phenotype and five gene pairs have been observed to induce a synthetic rescue phenotype.  Figure 3 indicates which double-gene knockouts are correctly predicted using the dynamic yeast cell cycle model. Seven of thirteen synthetic lethal and two of five synthetic rescue phenotypes were correctly predicted. A significant disparity is observed between the accuracy of prediction of single gene compared to double-gene knockouts. The relatively poor prediction of double-gene knockouts may be a result of the size of the model. It can be assumed that many of the interactions related to cell viability in yeast cannot be represented in our seventeen gene model. 

### 2.3. Investigation of the Model Interaction Space Identifies Consensus Models

**Figure 4:**
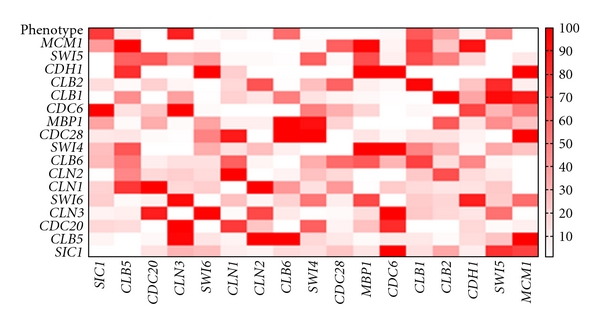
Heat map indicating the frequency of input gene selection in the top 100 best-fit rules for each output gene and fraction of budding cells. Input genes are ordered along the horizontal axis. Output genes are ordered along the vertical axis. The color of a square *i*,* j* represents the frequency with which input gene *i *is observed among the top 100 best-fit rules for output gene (or phenotype) *j*.

Given the use of an exhaustive search through the model parameter space, analysis of the best fit models alone neglects a significant portion of the data. We have previously demonstrated that the number of acceptable alternative models decreases exponentially with increasingly stringent fitness thresholds [9]. Analysis of a small number of alternative, approximately equally fit models may provide additional insight into the biological phenomena under investigation. With this in mind, we examined the input genes selected in the top hundred best fitting models for each gene and the fraction of budding cells (Figure 4). Most of the models for a given output are largely consistent, with a small number of inputs selected in a majority of best fit models. Input genes that appear in a majority of alternative models are more likely to influence output. For example, *CDC6 *is an input gene in the majority of best fit models for *SIC1 *expression. This reflects the strong coexpression and coregulatory relationships between *CDC6* and *SIC1* [24, 25]. Input genes that appear in a small number of the alternative models (e.g., *Swi4 *in the model for *SIC1*) likely represent noise or may have only a small influence on output. It is also interesting to note the inconsistencies between the best fit models and the alternative models. For example, *MBP1 *is more frequently observed as an input to the *Swi5* model than *CDH1* and *Swi6,* but *MBP1* is not a factor in the best fit model. These genes may represent alternative hypotheses that can be examined by further experimentation.

## 3. Discussion 

In this paper, we describe the development and analysis of a fuzzy logic model of cell cycle in *Saccharomyces cerevisiae* relating the expression of seventeen cell cycle genes to the budding phenotype. The structure of the model and semi-quantitative rules describing regulatory interactions between genes and between genes and phenotype were derived from a time series gene expression microarray dataset using an exhaustive search method. Best fit models for each gene and phenotype were identified and interpreted based on agreement with known interactions from the literature. In addition, node-specific models were integrated into a composite network model, and a simple iterative scheme was used to approximate the dynamic behavior of the system. The dynamic model converges to two alternative self-consistent states, matching hypotheses developed from experimental investigation. The composite network model was then analyzed to identify essential genes, that is, genes which are necessary for viability, and to predict synthetic lethal and synthetic rescue phenotypes *in silico*. This work represents a proof of concept demonstrating the feasibility of integrating phenotype information into mechanistic transcriptional models and the value of this approach in guiding hypothesis generation.

Yeast cell cycle was chosen as a model system to evaluate our method because it is a relatively well-characterized process with extensive literature and datasets available for model generation and evaluation. Association of model predictions with prior knowledge demonstrates the capability of the fuzzy logic model generation process to infer biologically relevant interactions. Note that genes which are expressed in the same phase of the cell cycle tend to be linked by positive interactions (e.g., G1 cyclins: *CLN1*-*2*, *CLB5-6, CLB5-CLN2*; M phase genes: *SWI5*, *CLB1*, *CLB2*; M/G1 transition genes: *CDC6*, *SIC1*, *SWI4*) [15, 18]. These edges represent coexpression relationships. Coregulatory relationships are also represented. For example, positive interactions between the *Swi5*, *CLB1*, and *CLB2* genes are indicative of coregulation by the *MCM1*/*XBP1* transcription factor complex [26]. Finally, functional relationships are also inferred. For example, *CLN1 *and *CLN2 *stimulate their own expression through a positive feedback loop and also up-regulate *CLB5/CLB6 *[27]. The model relating expression of the *SIC1*, *CLB1*, *CLN3*, *CLB6,* and *Swi5 *expression to the fraction of budding cells also captures known biological relationships. Negative regulation of the budding phenotype by *SIC1* corresponds with the inhibitory function of *SIC1* on *CDC28*/B-type cyclin complex formation and G1/S phase transition [28]. Interactions between the fraction of budding cells and *CLB6*, *CLB1*, *CLN3, *and *Swi5 *reflect coexpression relationships. *Swi5 *and *CLB1* are expressed in S, G2, and M phase when budding occurs [18]. *CLB6 *and *CLN3 *are active in G1 phase when the budding phenotype is dormant. Confirmation of model predictions with known biological information suggests that the fuzzy logic modeling framework accurately captures relationships between genes and between gene expression and phenotype. 

*In vitro* gene knockout is a fundamental method in molecular biology to characterize gene function by identifying genes that are necessary for cell viability and by inferring gene-gene interactions. We integrated the gene and phenotype-specific fuzzy logic models into a composite network model in order to investigate the dynamic behavior of the model. Investigation of the expression of genes and the predicted fraction of budding cells at convergence of the dynamic model indicates the presence of multiple local minima, associated with a high, medium, and low fraction of budding cells. Next, we used the dynamic model to predict the viability of gene deletion mutants *in silico*. Large collaborative efforts have systematically surveyed the effect of single gene deletions in model organisms such as *Saccharomyces cerevisiae* using molecular barcodes to quantify the fitness of gene deletion mutants grown together in culture [14]. This data provides a rich test dataset in order to validate the predictions generated by the fuzzy logic model (Table 3). In total, the viability of fourteen out of seventeen single gene deletion mutants was correctly predicted. We correctly predicted that deletion of the *MCM1*, *CDC28,* and *CDC20 *genes are associated with an inviable phenotype. All of these genes are known to have important roles in cell cycle progression. *CDC28* is the most active cyclin-dependent kinase in the yeast genome [18, 29], facilitating cell cycle progression through protein-protein interactions with cyclins expressed at different stages of the cell cycle. *MCM1 *is a transcription factor that interacts with other transcriptional cofactors to regulate expression of multiple mitotic genes including *CLB1, CLB2, Swi4, Swi5, CLN1, CLN3,* and* CDC6* and is essential in regulating the G2/M phase transition [26, 30–33]. *CDC20 *targets the *Pds1* gene for ubiquitin-mediated degradation and forms a complex essential for G2/M phase check-point function [18]. Accurate prediction of the viability of single gene deletions in higher organisms may improve the rate at which gene function can be characterized in the context of the transcriptional regulatory network. 

As an additional challenge, we tested the ability of the dynamic cell cycle model to predict synthetic lethal and synthetic rescue double mutant phenotypes. Nine out of eighteen double-mutants were correctly predicted (Figure 3). Incorrect single-gene and double-gene knock-out phenotype predictions may be a result of the limited scope of our model. For example, a synthetic lethal phenotype is observed experimentally with knockout of the *CLB5 *and *Swi4 *genes [34] but not predicted by our model. Cells with the *CLB5/Swi4 *knock-out arrest at the G2/M phase transition. Arrest is attributed to activation of the DNA damage checkpoint dependent on expression of the *RAD9* and *RAD24* genes [34]. These results suggest that the incorporation of data related to the DNA damage response may be necessary to correctly predict the phenotype of the *CLB5/Swi4* knockout. In a similar manner, use of additional gene and phenotype data may improve the prediction of double-gene knockouts. 

Although fuzzy logic offers a robust, interpretable and computationally efficient modeling methodology, there are a number of limitations to the approach we have described. Fuzzy logic is associated with an inherent curse of dimensionality problem that arises from the evaluation and combination of individual rules that effect nodes with multiple inputs. In an exhaustive search, the number of potential models that must be evaluated grows exponentially *O*(*m* ^*N*^), in which *N* represents the number of inputs and *m* represents the number of possible rules relating the effect of input on the output. We limited the exhaustive search by bounding the solution space to rules that incorporated five or less inputs. This approximation of gene regulation likely limits the accuracy with which we can reproduce biological mechanisms as the expression of many genes appears to be controlled by more than five inputs. Other solutions to the curse of dimensionality issue could involve the incorporation of optimization methods to allow for a directed search of the parameter space. As an example, an alternative method utilizing genetic algorithms to identify best fit models has been developed [8]. Evaluation of the directed search algorithm indicated that the best fit solution as determined from an exhaustive search is reached in 98% of simulations with much less computational time. Importantly, use of optimization algorithms would allow for the investigation of systems with a larger number of genes and phenotypes without an explicit constraint on the number of inputs. In addition, the approach we have described cannot easily incorporate statistics to estimate the significance of model fit. As an alternative, we compared model predictions to prior knowledge and observations from independent experiments in order to evaluate the generalizability of our model. Finally, there are no formalized methods for defining the parameters involved in building a fuzzy logic system, such as the number of fuzzy sets and the shape of membership functions. These parameters can be selected with the use of prior knowledge or by using heuristics, but these approaches are less than optimal. These shortcomings should be considered when determining whether a fuzzy logic method similar to the implementation we have described is appropriate for future applications. 

The explicit incorporation of phenotype as a node in the molecular signaling model is a key aspect of this work that allows for prediction of the viability of gene knockout *in silico* and hypothesis generation. A number of other studies have included phenotype in the inference of molecular signaling networks for similar purposes [1, 4, 6]. For example, in a series of publications, investigators have developed a decision tree framework to relate the expression of signaling proteins to migration speed of fibroblasts and breast cancer cells under different conditions [1, 4]. The goal of decision tree analysis is to generate a predictor capable of discriminating between multiple classes (e.g., low, medium, high speed of migration) with maximum accuracy and minimum model complexity. Using the model, investigators predicted the effect of modifications to the substrate surface and the addition of growth factors on migration speed with 70% accuracy [1]. In addition, investigators correctly predicted the stimulatory effect of MLCK inhibitors on migration speed [4]. In a second example, investigators used a partial least squares approach to build a model relating various metrics capturing the expression, activity, and/or phosphorylation of 19 molecular species to a signature of apoptotic activity in human colon adenocarcinoma cells [6]. Partial least squares is similar to principal component analysis in that it involves the decomposition of data into orthogonal projections that capture a majority of the information in the original dataset. However, in partial least squares analysis, projections are selected to maximize the covariance between independent (e.g., expression, activity and/or phosphorylation of molecular species) and dependent (e.g., apoptotic signature) variables. In this study, investigators used partial least squares to identify a three-dimensional model capable of predicting the state of apoptosis outputs with 94% accuracy. Further analysis indicated that a majority of model accuracy could be attributed to two principal components associated with a stress-apoptosis response mediated through *JNK1* and *MK2* activity and cleavage of caspase 8 and a prosurvival response mediated through phosphorylation of *Akt*, *IRS1*, *FKHR*, and procaspase 3 metrics. While these approaches have successfully identified relations between gene expression and phenotype, it should be noted that the experimental methods used to gather information in these studies, including kinase activity assays, antibody arrays, western blots, and immunoblotting [1, 4, 6], are relatively low throughput. We have demonstrated the value of our model using gene expression microarray data, which facilitates collection of data at a genome-wide scale. In this study, we use a bounded exhaustive search that limits the size of the modelled system. Replacing the exhaustive search with an optimization method, as described above, would allow us to model larger systems and take advantage of the higher throughput of gene expression microarrays. More importantly, decision trees and partial least squares generate abstract representations of the underlying biological network, while in the present model, we use expression data to determine the network topology based on gene expression. We contend that the fuzzy logic and optimization framework we have described provides an optimized depiction of transcriptional networks in the cell, necessary to gain a thorough understanding of the mechanisms that contribute to changes in phenotype. 

Use of gene expression microarray data for the generation of mechanistic signaling models has advantages and disadvantages. Gene expression microarrays enable cost-effective measurement of transcript abundance at the genome-wide scale. Widespread use of gene microarrays for expression profiling has led to the development of robust algorithms for preprocessing [35–37], quality control [36], data visualization [38], and analysis [39, 40]. In addition, guidelines and infrastructure have been established to promote the sharing of gene expression microarray data [41]. However, by using gene expression data for model generation, we are admittedly neglecting posttranscriptional regulatory events, such as posttranslational modifications, protein-protein interactions, and protein degradation. Accordingly, many of the relationships we have derived are indicative of coexpression or coregulation at the transcriptional level. As the proteomics field matures, it will become more feasible to incorporate information regarding posttranscriptional regulatory mechanisms into biological network models. In addition, the dataset we have used in this study was collected on two-color cDNA technology. Two-color cDNA arrays are outdated in comparison to commercial oligonucleotide arrays. Despite this caveat, there is nothing in the literature that suggests the data is incorrect, and it is one of the only published datasets that provides both transcriptional and phenotypic data required for our algorithm. 

The majority of the analysis presented in this work is based on the best fit approximations (Figure 1) to gene expression data. In previous work, we demonstrated that the number of acceptable alternative models decreases exponentially with error tolerance [9]. The exhaustive search method generally converges to a small number of equally likely alternative models with high fitness to experimental data. Investigation of the interactions selected in the top hundred best fitting models for each output provided the means to examine these alternative models. In some cases, alternative models represent similar relationships (e.g., the following rules relating *CLN1 *to *CDC20 *expression: {3  2  1}, {2  2  1}). These models can be reasonably merged into a consensus model in which expression of *CLN1* inhibits expression of *CDC20*. In other instances, alternative models may indicate parallel regulatory pathways that are activated under different conditions. Finally, alternative models may offer different or even contradictory interpretations. Models that fit the latter description represent alternative hypotheses that are equally supported by the available data. Incorporation of other types of data may provide additional evidence to reject false positive models. For example, ChIP on chip data could provide additional evidence to support the identification of gene regulatory interactions [42, 43]. ChIP on chip combines chromatin immunoprecipitation (ChIP) with microarray analysis to map the genome-wide location of protein-DNA binding sites. This methodology can be used to infer the gene targets of a transcription factor of interest and, therefore, experimentally validate coregulatory interactions inferred by the model. As another example, prior knowledge in the form of known interactions could be incorporated into the model generation process. In this case, it will be important to limit the influence of prior knowledge such that discoveries that are strongly supported by the data will not be rejected. Alternatively, identification of contradictions in model predictions can be used to generate novel hypotheses and design experiments in order to gather the data necessary to reject incorrect models.

## 4. Conclusions 

Molecular profiling technologies such as gene expression microarrays have enabled the quantification of molecular abundance at a genome-wide scale in high throughput. As technologies have matured, goals of data analysis have grown from the identification of conserved expression patterns across samples or conditions to the comprehension of gene function in the context of complex regulatory and functional networks. In this study, we have extended a fuzzy logic-based modeling approach to derive a transcriptional network consisting of seventeen genes known to be important for cell cycle regulation in yeast and a network element representing a phenotypic observation (the fraction of budding cells). Both the topology (i.e., interactions) and regulatory traits (e.g., stimulation, inhibition) of relationships between genes and between genes and phenotype are derived from publicly available gene microarray expression data and phenotype data using a bounded exhaustive search of the potential interaction space. Comparison of inferred gene regulatory interactions with known interactions in the literature provides confidence in the biological relevance of model predictions. Through the analysis of best fit fuzzy logic signaling models, genes with direct and indirect effects on phenotype were identified. In addition, we used our model to predict the effects of gene knockdown on cellular viability. In this manner, the methodology we have developed provides a direct link between computational analysis of molecular profiling data and experimental observations. We envision coupling this computational modeling method with experimentation in an iterative fashion to incrementally build understanding of regulatory mechanisms that control poorly understood cellular functions. This approach allows for systematic characterization of gene function in the context of disease-related, functional mechanisms that will facilitate rational design of targeted therapies.

## 5. Methods

### 5.1. Gene Expression and Phenotype Data

Gene expression microarray data and phenotype data were obtained from the Yeast Cell Cycle Analysis Project at the Stanford University (http://genome-www.stanford.edu/cellcycle/) [15]. Gene expression was measured using in-house two-color microarrays as described [15]. The fraction of budding cells was determined by manually scoring and counting budding cells. 

### 5.2. Preprocessing of Gene Expression Data

Expression data was projected onto the interval [−1,1] for generality and compatibility with the fuzzy logic scheme described below. Expression ratios were preprocessed by log base 2 transformation followed by normalization using the arctangent function and division by *π*/2. This process results in a symmetric transformation of the data across the desired interval.

### 5.3. Fuzzy Logic System

A fuzzy logic framework [9] was used to derive a model of the regulatory relationships between 17 cell-cycle-related genes and the influence of gene expression on the fraction of budding cells. The model can be described as a directed graph, in which the nodes represent genes or phenotypes and the edges represent direct or indirect interactions. Phenotype information was integrated by including a node for the fraction of budding cells and identifying best fit models in which gene expression could be used to predict phenotype. The budding network model was generated by joining independent models of each node that were derived through application of the following steps: fuzzification, rule configuration, defuzzification, and evaluation. 

**Figure 5:**
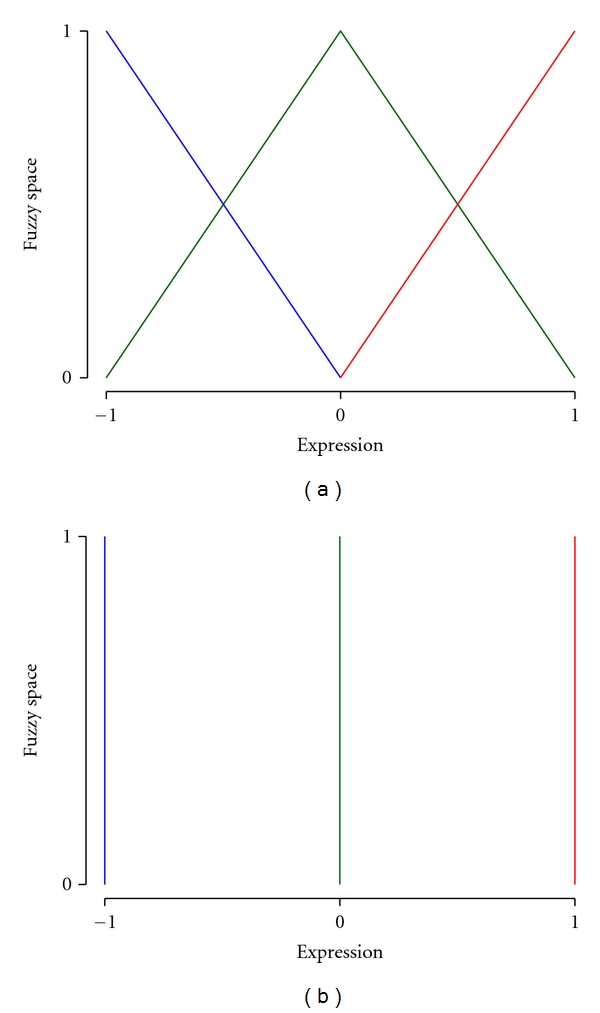
Membership function and defuzzification function used for converting gene expression values from continuous to fuzzy space and back. (a) Membership function describing the transformation of gene expression values into three fuzzy sets of low (blue), medium (green), and high (red) expression. (b) Point set definitions for defuzzification of fuzzy gene expression values via the simplified centroid method.

Fuzzification utilizes membership functions to convert continuous measurements into a discrete representation. The range of the membership function represents the degree to which a value belongs to a fuzzy set. The choice of membership function is subjective and context dependent. In the framework described here, we employ a membership function (Figure 5(a)) consisting of three fuzzy sets (low, medium, and high expression). This membership function was selected to maximize computational efficiency, exactly reproduce monotonic linear negative and positive interactions and avoid the introduction of systematic errors through fuzzification. Given three fuzzy sets (*y*_1_ = low, *y*_2_ = medium, *y*_3_ = high), fuzzification of a gene expression value *x *results in the generation of a fuzzy set **y **= [*y*_1_, *y*_2_, *y*_3_] as follows:

(1) $$\begin{array}{cc} & y_{1}=\{\begin{array}{cc} -x, & x<0,\\ 0, & x\geq 0, \end{array}\\ & y_{2}=1-\left|x\right|,\;\forall x,\\ & y_{3}=\{\begin{array}{cc} 0, & x\leq 0,\\ x, & x>0. \end{array} \end{array}$$

The following example demonstrates how normalized gene expression values from three different genes (*x*_*g*1_, *x*_*g*2_, and *x*_*g*3_) are represented in fuzzy space (*y*):

(2) $$\begin{array}{c} \begin{array}{c} x=\left\{x_{g1}\;x_{g2}\;x_{g3}\right\}=\left\{0.684\;0\;-0.125\right\},\\ y=\left\{\left[y_{1}^{x_{g1}}\;y_{2}^{x_{g1}}\;y_{3}^{x_{g1}}\right]\;\left[y_{1}^{x_{g2}}\;y_{2}^{x_{g2}}\;y_{3}^{x_{g2}}\right]\;\left[y_{1}^{x_{g3}}\;y_{2}^{x_{g3}}\;y_{3}^{x_{g3}}\right]\right\}, \end{array}\\ y=\left\{\left[0\;0.316\;0.684\right]\;\left[0\;1\;0\right]\;\left[0.125\;0.875\;0\right]\right\}. \end{array}$$

A similar scheme could be used for fuzzification of phenotype data. In this study, phenotype is considered as an output only. Accordingly, it is not necessary to fuzzify the observed fraction of budding cells. However, model predictions are defined in fuzzy space. In order to evaluate and interpret model predictions, it is necessary to defuzzify the predicted fraction of budding cells. Defuzzification schemes are dependent on membership functions. With this in mind, the membership function for fuzzification of the fraction of budding cells is described as follows, for completeness:

(3) $$\begin{array}{c} y_{1}=\{\begin{array}{cc} 1-2x, & x<0.5,\\ 0, & x>0.5, \end{array}\\ y_{2}=1-\left|2x-1\right|,\;\forall x,\\ y_{3}=\{\begin{array}{cc} 0, & x<0.5,\\ 2x-1, & x>0.5. \end{array} \end{array}$$

Through fuzzification, continuous data is discretized to increase computational efficiency in the rule configuration stage. Importantly, information needed to derive qualitative relationships between nodes remains observable in the fuzzy data. 

Rule configuration is the specification of if-then relationships between variables in fuzzy space. For example, an inhibitory relationship is represented by the rule vector **r** = [*r*_1_ *r*_2_ *r*_3_] = [3  2  1] (i.e., if input is low (*r_1_*), then output is high (3); if input is medium (*r_2_*), then output is medium (2), etc.). The state of an output node **z **= [*z_1_ z_2_ z_3_*] is determined by the fuzzy state of an input gene **y **= [*y_1_ y_2_ y_3_*] and the rule describing the relation from input to output **r** = [*r*_1_ *r*_2_ *r*_3_] as follows:

(4) $$z=\left[y_{r_{1}}\;y_{r_{2}}\;y_{r_{3}}\right].$$

Extending the example given above, assume that expression of gene 1 inhibits or negatively regulates the output gene. Given the expression of gene 1 and a rule describing the relationship between gene 1 and the output gene, the value of **z** in fuzzy space can be predicted as follows:

(5) $$\begin{array}{c} y_{g1}=\left[0\;0.316\;0.684\right],\\ r_{g1:z}=\left[3\;2\;1\right],\\ z=\left[0.684\;0.316\;0\right]. \end{array}$$

In biological networks, the state of output nodes is generally dependent on multiple input nodes. By convention, multiple inputs are integrated in fuzzy logic using the logical AND connective (if gene 1 is low and gene 2 is high, then the output gene is low). This leads to a combinatorial rule explosion in which the addition of inputs to the fuzzy logic system causes an exponential increase in the number of rules to be evaluated and computational time [44]. Alternatively, the relation of each input to the output can be evaluated separately and joined using the logical OR connective. This alternative rule configuration is equivalent to the logical AND rule configuration in propositional logic [44]. Furthermore, the addition of inputs to the fuzzy logic system under the alternative configuration results in a linear increase in computational time. The logical OR connective, or union, can be interpreted as the algebraic sum in fuzzy logic [12, 13]. Under this interpretation, rules describing the relation of each input gene to the output gene are evaluated separately resulting in intermediate outputs (*z^i^*). The intermediate outputs are summed to determine the expression of the output gene (*z*) in fuzzy space:

(6) $$z=\overset{N}{\underset{i=1}{\sum }}z^{i}=\left[\overset{N}{\underset{i=1}{\sum }}z_{1}^{i}\;\overset{N}{\underset{i=1}{\sum }}z_{2}^{i}\;\overset{N}{\underset{i=1}{\sum }}z_{3}^{i}\right],$$

where *N* is equal to the number of input genes that regulate the expression of the output gene. The example below demonstrates the application of this rule configuration given that gene 1 (*y*_*g*1_) negatively regulates the output gene and gene 3 (*y*_*g*3_) positively regulates the output gene as follows:

(7) $$\begin{array}{c} \begin{array}{c} \begin{array}{c} y_{g1}=\left[0\;0.326\;0.684\right],\;\;y_{g3}=\left[0.125\;0.875\;0\right],\\ r_{g1:z}=\left[3\;2\;1\right],\;\;r_{g3:z}=\;\left[1\;2\;3\right], \end{array}\\ z_{g1:z}=\left[0.684\;0.326\;0\right],\;\;z_{g3:z}=\left[0.125\;0.875\;0\right], \end{array}\\ z=\left[0.809\;1.206\;0\right]. \end{array}$$

The complexity of the rule configuration step is dependent on the number of possible rules describing relations between nodes (with three fuzzy sets, the number of rules = 3^3^ = 27) and the number of input genes for a given node. In this work, we limit the number of input genes to less than or equal to five to bound the computational cost. 

Defuzzification is the inverse transformation of variables from fuzzy representation to continuous space. This step is necessary prior to evaluation of model predictions against independent test data. Predicted expression values $\left(\tilde{x}\right)$ defined in continuous space can be derived from discrete, fuzzy values via the simplified centroid method with point set definitions as shown in Figure 5(b) [13]. Given the fuzzy values of a gene **y** = [*y_1_ y_2_ y_3_*], the defuzzified expression value $\left(\tilde{x}\right)$ can be defined as

(8) $$\tilde{x}=\frac{y_{3}-y_{1}}{y_{1}+y_{2}+y_{3}}.$$

The location of the centroids in the simplified centroid method is dependent on the location of the apex of the corresponding membership functions used in fuzzification. With this in mind, defuzzification of phenotype values is accomplished by a similar function with the point set definitions shifted to reflect the shift in the centroids of membership functions defined in (3). 

Following defuzzification, expression measures are reverse transformed back to log_2_ expression values by multiplying by *π*/2 and applying the tangent function. Similarly, the defuzzified fraction of budding cells should be transformed to remove the sigmoidal bias that originates from arctangent transformation of the gene expression values. The fraction of budding cells is defined on the interval [0,1]. Accordingly, phenotype measurements were reverse transformed using the logistic function:

(9) $$y\left(x\right)=\frac{1}{1+e^{-Cx}},$$

where *C* is equal to 6 and the independent variable *x* on the right side of (9) is set equal to (2*x* − 1). Parameter values of the logistic function were selected to evenly distribute the transformed data over the range of [0,1].

Best fit models describing the relationships between genes and between genes and the fraction of budding cells were generated using an exhaustive search through the parameter space. For a given output node, all combinations of input genes and rules were evaluated by comparing the predicted expression value against the experimental data. The coefficient of determination (*R* ^2^) metric was used for comparison. The coefficient of determination is based on the Euclidean distance between the predicted and observed values. Models with high coefficient of determination accurately predict the magnitude of expression. Models analyzed in this study represent the best fit to the data as determined using this metric.

### 5.4. Incorporating Redundancy in the Model for Phenotype

In the fuzzy logic framework described above, each input rule affects the state of associated output nodes through separate, independent relations. However, biological pathways are often populated with redundant mechanisms. To obtain a model that accurately represents the underlying biology, these homologous relationships must be incorporated. We have manually modified the inferred model to represent pairs of functionally homologous genes as the maximum of the two expression values. All instances of homologous gene pairs (*CLN1 *and *CLN2*, *CLB1* and *CLB2*, *CLB5* and *CLB6*) in the input of gene or phenotype modes were modified in this manner. For example, the model for the fraction of budding yeast phenotype was modified as described below:

inferred model: *CLN3* & *CLB6* & *CLB1* & *SWI5* & *SIC1⇒* phenotype,
modified input: *CLN3* & (*CLB5/CLB6*) & (*CLB1/CLB2*) & *SWI5* & *SIC1⇒* phenotype,
where, (*CLB5/CLB6*) = Max(*CLB5*, *CLB6*) (*CLB1/CLB2*) = Max(*CLB1*, *CLB2*).

### 5.5. Analysis of the Network Model

Given the inferred models for each node, a composite network model was generated to investigate the behavior of the system as a whole. Node-specific models were integrated into a single global model by consolidating all instances of a gene into a single node. An iterative scheme was employed to determine the state of the network at equilibrium. Experimentally observed values of gene expression were used as initial conditions (*I_0_*) for the state of the corresponding nodes. New values of each node (*I_1_*) were calculated based on the initial conditions and the fuzzy relations inferred from the data. Values in the next iteration were calculated as a linear combination of the inferred values (*I_n_*) and the initial values (*I_n-1_*) as follows:

(10) $$I_{n+1}=\alpha I_{n}+\left(1-\alpha \right)I_{n-1}.$$

Calculation of new values continued until the convergence condition of |*I*_*n*_ − *I*_*n*−1_| < 10^−7^ was reached. Linear combination of new and old values ensures that the system smoothly converges towards equilibrium. The value of the mixing parameter (**α**) can be chosen to minimize the number of iterations required to reach a solution while allowing for convergence. As the cost of the calculations was minimal, we chose a conservative value of 0.01 and found convergence could be reached after a few thousand iterations which required only a few seconds of simulation time. Larger choices of (**α**) could decrease the number of iterations but can also cause the algorithm to diverge from the true solution.

**Computational Implementation**

Model building and the exhaustive search was implemented in Fortran. Dynamic network simulations were implemented in Matlab.

## Supplementary Material

Supplementary Table 1: Goodness of fit of predicted values versus observed expression for the best fitting models. The goodness of fit of the original gene-centric models are listed in column 3. The goodness of fit of models with homologous gene approximations are listed in column 4.

## Figures and Tables

**Table 1:** Canonical functions of modelled genes in cell cycle regulation.

	*CLN1*	G1 cyclin: activates *CDC28* kinase to promote the G1 to S phase transition; *CLN-CDC28* complex enhances ubiquitin-mediated proteolysis of *SIC1* by phosphorylation, regulates START-related events: budding, spindle-pole-body duplication, and DNA synthesis, and homologous with *CLN2 *
	*CLN2 *	G1 cyclin: activates *CDC28* kinase to promote the G1 to S phase transition; *CLN-CDC28* complex enhances ubiquitin-mediated proteolysis of *SIC1* by phosphorylation, regulates START-related events: budding, spindle-pole-body duplication, and DNA synthesis, and homologous with *CLN1 *
	*CLN3 *	G1 cyclin: activates *CDC28* kinase to promote the G1 to S phase transition and regulates transcription of *CLN1* and *CLN2 *
Cyclins	*CLB1 *	G2 cyclin: activates *CDC28* kinase to promote the transition from G2 to M phase, promotes spindle elongation during mitosis, negatively regulates SBF-mediated transcription, and homologous with *CLB2 *
	*CLB2 *	G2 cyclin: activates *CDC28* kinase to promote the transition from G2 to M phase, promotes spindle elongation during mitosis, negatively regulates SBF-mediated transcription, and homologous with *CLB1 *
	*CLB5 *	G1/S cyclin: activates *CDC28* kinase to promote initiation of DNA synthesis, inactivated by *SIC1*, negatively regulates *CLN-CDC28* complex formation, forms mitotic spindles in association with *CLB3* and *CLB4*, and homologous with *CLB6 *
	*CLB6 *	G1/S cyclin: activates *CDC28* kinase to promote initiation of DNA synthesis, inactivated by *SIC1*, negatively regulates *CLN-CDC28* complex formation, forms mitotic spindles in association with *CLB3* and *CLB4*, and homologous with *CLB6 *

*CDC*	*CDC6 *	ATP-binding protein required for DNA replication, component of the pre-replicative complex (pre-RC) which is required for *MCM2-7* DNA association
	*CDC28 *	Cyclin-dependent kinase: alternately associates with G1 cyclins and G2/M cyclins, regulates spindle-pole-body duplication, budding, *SIC1* proteolysis in association with *CLN1-3*, regulates DNA replication in association with *CLB5-6*, and regulates spindle assembly, inactivation of *CLN* transcription through repression of SBF transcription factor in association with *CLB1-2 *

	*MCM1 *	Regulates expression of *CLB1*, *CLB2*, *BUD4*, *SWI5* in M phase: *CLN3*, *SWI4*, *CDC6* at M/G1 transition
	*SWI4 *	Transcription cofactor, forms complex with *SWI6* and regulates transcription of *CLN1-2*; activity initiated by *CLN3-CDC28*, repressed by *CLB2-CDC28 *
Transcription factors	*SWI5 *	Activates transcription of *SIC1* and *CDC6* at the M/G1 phase boundary and in G1 phase; localization to the nucleus occurs during G1, regulated by *CDC28*-mediated phosphorylation
	*SWI6 *	Transcription cofactor: forms complexes with DNA-binding proteins *SWI4* and *MBP1* to regulate transcription at the G1/S transition and regulates transcription of *CLN1-2*, *CLB5-6*; localization regulated by phosphorylation; transcriptional activity initiated by *CLN3-CDC28* complex, repressed by *CLB2-CDC28* complex
	*MBP1 *	Transcription cofactor: forms complex with *SWI6* and regulates transcription of *CLB5-6 *

Inhibitors	*SIC1 *	Inhibits *CLB5/6-CDC28* complex formation, inhibits G1/S transition, downregulates *CLB-CDC28* activity in late stages of mitosis, targeted for ubiquitin-mediated proteolysis by *CLN-CDC28 *
	*CDH1 *	Activates anaphase promoting complex/cyclosome (APC/C) and directs ubiquitinylation of cyclins, cell division cycle genes
	*CDC20 *	Activates anaphase-promoting complex/cyclosome (APC/C) and directs ubiquitination of *CLB1-4*, *PDS1 *

**Table 2:** Goodness of fit of predicted versus observed expression for the best fitting models. The fuzzy models that produce these metrics are displayed in Figure 1.

Node	ORF	COD
*SIC1*	YLR079W	0.8524
*CLB5*	YPR120C	0.9484
*CDC20*	YGL116W	0.8270
*CLN3*	YAL040C	0.8296
*SWI6*	YLR182W	0.8128
*CLN1*	YMR199W	0.9643
*CLN2*	YPL256C	0.9314
*CLB6*	YGR109C	0.8431
*SWI4*	YER111C	0.8659
*CDC28*	YBR160W	0.6770
*MBP1*	YDL056W	0.7485
*CDC6*	YJL194W	0.8485
*CLB1*	YGR108W	0.9322
*CLB2*	YPR119W	0.9316
*CDH1*	YGL003C	0.7956
*SWI5*	YDR146C	0.9262
*MCM1*	YMR043W	0.7745
Phenotype	NA	0.7264

**Table 3:** *In silico* gene knock-down models predict the viability of experimental deletion of yeast cell cycle genes. Outcome of gene deletion experiments obtained from the *Saccharomyces* Genome Deletion Project [20]. Viability of *in silico* gene knock-down models assessed by the fit of the predicted to the observed fraction of budding cells. 14/17 predictions are correct. Incorrect predictions are marked in red.

Node	ORF	Experimental outcome	Model prediction
*SIC1*	YLR079W	Viable	Viable
*CLB5*	YPR120C	Viable	Viable
*CDC20 *	YGL116W	Inviable	Inviable
*CLN3*	YAL040C	Viable	Viable
*SWI6*	YLR182W	Viable	Viable
*CLN1*	YMR199W	Viable	Viable
*CLN2*	YPL256C	Viable	Viable
*CLB6*	YGR109C	Viable	Viable
*SWI4*	YER111C	Viable	Viable
*CDC28*	YBR160W	Inviable	Inviable
*MBP1*	YDL056W	Viable	Viable
*CDC6*	YJL194W	Inviable	Viable
*CLB1*	YGR108W	Viable	Viable
*CLB2*	YPR119W	Viable	Viable
*CDH1*	YGL003C	Viable	Inviable
*SWI5*	YDR146C	Viable	Inviable
*MCM1*	YMR043W	Inviable	Inviable
