# Supplementary material for: An Integrated Framework to Model Cellular Phenotype as a Component of Biochemical Networks
Source: Adv Bioinformatics. 2011 Nov 29;2011:608295. doi: 10.1155/2011/608295 (PMC3235418; doi:10.1155/2011/608295)
Supplement: Supplementary file 1 — Supplementary Table 1: Goodness of fit of predicted values versus observed expression for the best fitting models. The goodness of fit of the original gene-centric models are listed in column 3. The goodness of fit of models with homologous gene approximations are listed in column 4. [file 608295.f1.doc]

| **Node** | **ORF** | **W/out Homologs** | **Homologs** |
| --- | --- | --- | --- |
| SIC1 | YLR079W | 0.8524 | 0.8524 |
| CLB5 | YPR120C | 0.9484 | 0.9431 |
| CDC20 | YGL116W | 0.8270 | 0.7991 |
| CLN3 | YAL040C | 0.8296 | 0.7579 |
| SWI6 | YLR182W | 0.8128 | 0.8128 |
| CLN1 | YMR199W | 0.9643 | 0.8317 |
| CLN2 | YPL256C | 0.9264 | 0.9124 |
| CLB6 | YGR109C | 0.8431 | 0.7748 |
| SWI4 | YER111C | 0.8659 | 0.8168 |
| CDC28 | YBR160W | 0.6770 | 0.5307 |
| MBP1 | YDL056W | 0.7485 | 0.7224 |
| CDC6 | YJL194W | 0.8485 | 0.8485 |
| CLB1 | YGR108W | 0.9322 | 0.9189 |
| CLB2 | YPR119W | 0.9316 | 0.9221 |
| CDH1 | YGL003C | 0.7956 | 0.8143 |
| SWI5 | YDR146C | 0.9262 | 0.6996 |
| MCM1 | YMR043W | 0.7745 | 0.5814 |
| Phenotype | NA | 0.7264 | 0.7260 |
